# Engineering of Recombinant Human Papillomavirus 16 L1 Protein for Incorporation with *para*-Azido-*L*-Phenylalanine

**DOI:** 10.4014/jmb.2407.07033

**Published:** 2024-08-09

**Authors:** Jinhyeon Kim, Ki Jun Jeong, Geun-Joong Kim, Jong-il Choi

**Affiliations:** 1 Department of Biotechnology and Bioengineering, Chonnam National University, Gwangju 61186, Republic of Korea; 2 Department of Chemical and Biomolecular Engineering, KAIST, Daejeon 34141, Republic of Korea; 3 Department of Biological Sciences and Research Center of Ecomimetics, Chonnam National University, Gwangju 61186, Republic of Korea

**Keywords:** Human papillomavirus L1 protein, site-directed mutagenesis, *para*-azido-*L*-phenylalanine, viruslike particle, *Escherichia coli*

## Abstract

Human papillomavirus (HPV) L1 capsid protein were produced in several host systems, but few studies have focused on enhancing the properties of the L1 protein. In this study, we aimed to produce recombinant Human papillomavirus (HPV) L1 capsid protein containing *para*-azido-*L*-phenylalanine (pAzF) in *Escherichia coli*. First, we expressed the maltose-binding protein (MBP)-fused HPV16 L1, and 5 residues in HPV16 L1 protein were selected by the in silico modeling for amber codon substitution. Among the variants of the five locations, we identified a candidate that exhibited significant differences in expression with and without pAzF via genetic code expansion (GCE). The expressed recombinant MBP-HPV16L1 protein was confirmed for incorporation of pAzF and the formation of VLPs was tested *in vitro*.

## Introduction

Human papillomavirus (HPV) is known to cause cervical cancer in humans, and approximately 200 types of HPV have been identified [1, 2]. According to a 2019 World Health Organization (WHO) research, HPV infections contribute to 610,000 new cases of cervical cancer annually among women [3, 4]. Structurally, HPV is a T=7 icosahedral virus composed of the major capsid protein, L1, and the minor capsid protein, L2 [5-7]. L1 proteins can self-assemble into pentameric structures under physiological conditions, and 72 of these pentamers can further assemble into virus-like particles (VLPs) independent of the L2 proteins [8, 9]. VLPs expose epitopes on the L1 protein, triggering immune responses to produce antibodies against HPV, making the production of L1 proteins crucial for HPV vaccine development [10-12].

On the other hand, therapeutic and diagnostic proteins often have a short serum half-life due to proteolytic degradation upon injection into the body [13, 14]. To enhance their stability, studies have been conducted by improving their properties through various modifications, including albumination and PEGylation [14-16]. Notably, albumination offers an alternative to PEGylation, which can induce non-specific immune responses. For the albumination at specific sites of proteins, non-canonical amino acids (ncAAs) were incorporated into the protein, enabling precise conjugation [14, 17].

Genetic code expansion (GCE) is a representative method to incorporate ncAAs by constructing novel translation systems that do not cross-act with the host cell. This technique allows for the site-specific introduction of ncAAs with diverse structures and functional groups at specific amino acid positions using stop codons, quadruplet codons, or reassignment of sense codons [18-22]. For instance, ncAAs have been incorporated into therapeutic proteins such as adeno-associated viruses, human growth hormone, and interferons [21, 23, 24]. A recent study demonstrated that urate oxidase with *para*-azido-*L*-phenylalanine (pAzF) was expressed by orthogonal tRNA^CUA^/tyrosyl-tRNA synthetase (tyrRS) pair derived from *Methanococcus jannaschii* showed longer half-life than that without pAzF [25-29].

Previous studies have reported the expression of HPV L1 protein [30], but there has been limited investigation into modifying the protein, which can enhance the activity or prolong the half-life. In this study, recombinant HPV16 L1 protein variants with an amber codon were constructed by site-directed mutagenesis to incorporate pAzF into proteins. HPV16 L1 protein variants were expressed in *Escherichia coli* and purified. The purified HPV16 L1 protein was then evaluated for its ability to self-assemble into VLPs *in vitro*.

## Materials and Methods

### Strains and Growth Conditions

*Escherichia coli* BL21 (DE3) (New England Biolabs, USA) served as the host for gene cloning and expression. Briefly, the cells were cultured in Luria-Bertani (LB) medium supplemented with appropriate antibiotics (50 μg/ml ampicillin and 50 μg/ml chloramphenicol) at 18°C with shaking at 250 rpm for flask-scale cultures. For protein expression, 0.5 mM *para*-azido-*L*-phenylalanine (pAzF, Sigma-Aldrich, USA), 0.2% (w/v) *L*-(+)-arabinose, and isopropyl β-*D*-1-thiogalactopyranoside (IPTG, molar concentrations varied based on experimental conditions) were used.

### Screening for Amber Codon Substitution

The pGST-opt-HPV vector harboring the HPV16 L1 gene was previously reported [30]. Amino acid sequences were analyzed using AlphaFold2 to model the three-dimensional structure of the HPV16 L1 protein [31]. Based on structural analysis and existing literature, five specific locations (Y13, V47, F110, Y116, and F505) were selected for amber codon substitution. These sites were selected to avoid amino acids that could affect protein activity, antigen recognition, and self-assembly, and to exclude residues that are structurally buried or conserved within the HPV family. All structural models were produced and analyzed using PyMol [32].

### Vector Construction and Overexpression of MBP-HPV16L1 variants in Recombinant *Escherichia coli*

**Fig. 1:**
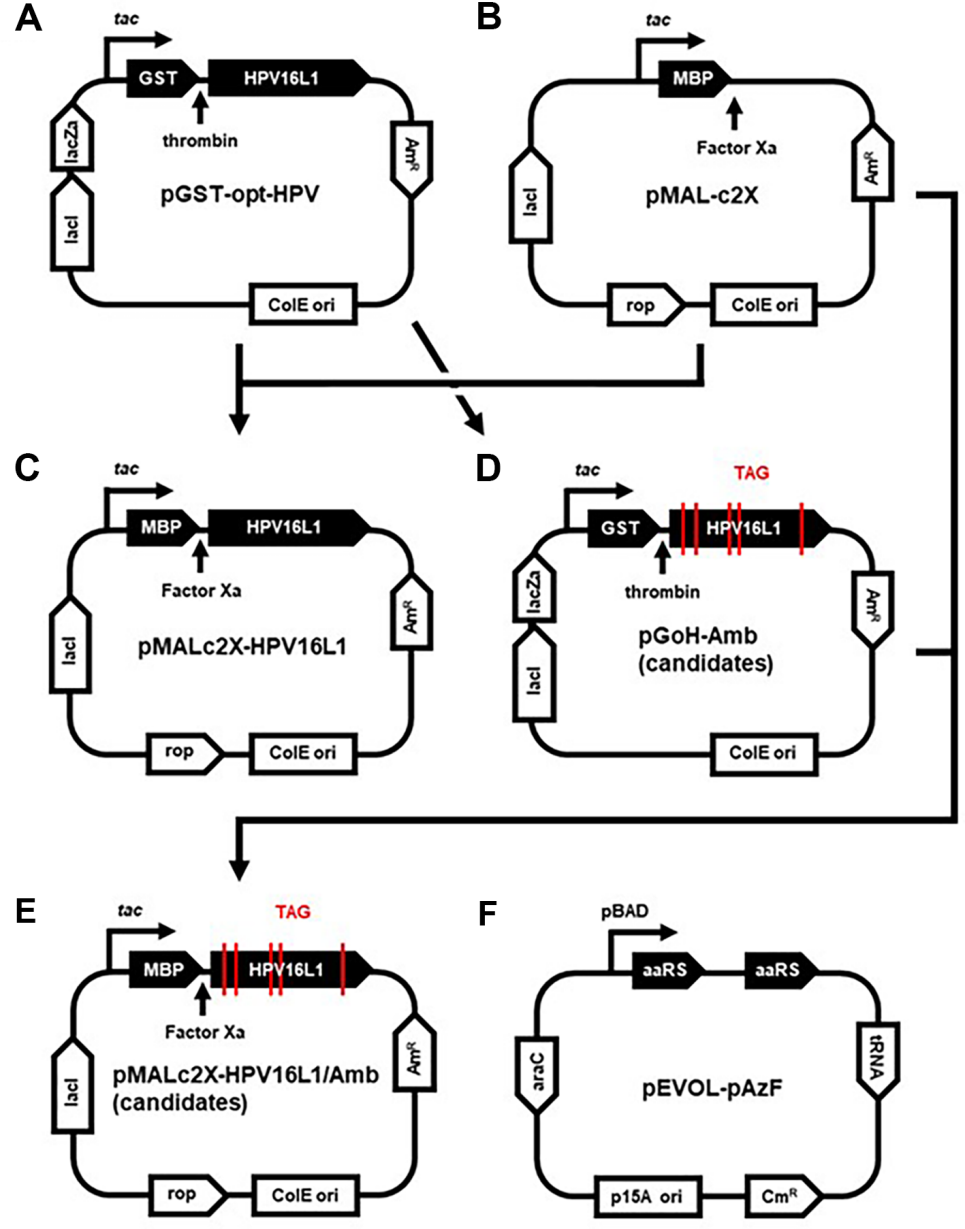
Schematic representation of the plasmids used in this study.

The HPV16 L1 gene in the pGST-opt-HPV vector (Fig. 1A) was amplified via a polymerase chain reaction (PCR) using the primers pMAL_HPV_FP1 and pMAL_HPV_RP1, and the backbone of the pMAL-c2X vector (Fig. 1B) was amplified using the primers pMAL_HPV_FP2 and pMAL_HPV_RP2. Phusion Plus DNA Polymerase (Thermo Fisher, Scientific) was used for PCR. The HPV16 L1 gene was then subcloned into pMAL-c2X to create a fusion protein with the MBP tag via Gibson assembly, yielding pMALc2X-HPV16L1 (Fig. 1C).

Subsequently, amber codon substitutions in the HPV16 L1 gene were performed through site-directed mutagenesis using the pGoH_Amb primers to construct pGoH-Amb vector candidates (Fig. 1D). Each of the five recombinant HPV16 L1 genes with the amber codon substitutions was amplified using the primers pMAL_HPV_sub_FP1 and pMAL_HPV_sub_RP1. Subsequently, they were individually subcloned into the pMAL-c2X vector as a fusion protein with the MBP tag via Gibson assembly, yielding pMALc2X-HPV16L1/Amb candidates (Fig. 1E).

The constructed plasmids were transformed into *E. coli* BL21 (DE3) via electroporation and selected on LB agar plates containing ampicillin. For the recombinant strains containing the amber codon-substituted HPV16 L1 gene, the pEVOL-pAzF plasmid (Fig. 1F) was additionally transformed and selected on LB agar plates containing both ampicillin and chloramphenicol. Detailed information regarding the plasmids, primers, and strains used in this study is provided in Table 1.

Five *E. coli* BL21 (DE3) pMALc2X-HPV16L1/Amb candidates with pEVOL-pAzF were grown with and without pAzF. These candidates were grown in LB medium containing ampicillin and chloramphenicol, and 4% of the seed culture was inoculated into 5 ml of fresh LB medium. When the OD_600nm_ reached approximately 0.6, IPTG was added to a final concentration of 0.1 mM, and the sample was incubated for 6 h to induce protein expression. The cells were then harvested, washed, and resuspended in 1× PBS (pH 7.4). Subsequently, the cells were sonicated, and the obtained lysates were analyzed via sodium dodecyl sulfate-polyacrylamide gel electrophoresis (SDS-PAGE).

### Confirming the Incorporation of pAzF into HPV16 L1

To confirm the incorporation of pAzF, DBCO-PEG_3_-FITC (Conju-Probe, USA) was conjugated with MBP-HPV16L1 F505γ. The expressed MBP-HPV16L1 F505γ was reacted with 10 μM of the DBCO-PEG_3_-FITC linker at room temperature (25°C, RT) for 1 h. The reaction samples were then visualized under UV light using a Quantum ST5 UV transilluminator (Vilber, France) [33].

### Purification of MBP-HPV16L1

To observe the self-assembly of the expressed HPV16 L1 proteins, recombinant *E. coli* BL21 (DE3) was grown in LB medium supplemented with ampicillin. A 2% inoculum of the seed culture was transferred into 50 ml of fresh LB medium. When the OD_600nm_ reached approximately 0.6, IPTG was added to a final concentration of 0.1 mM, and the sample was incubated for 6 h to induce protein expression. The cells were then harvested, washed, and resuspended in MBP Binding Buffer (200 mM NaCl, 20 mM Tris-HCl, 1 mM EDTA, pH 7.4). Subsequently, the cells were lysed via sonication, and the supernatant was obtained through centrifugation at 4,000 rpm for 30 min at 4°C. The obtained supernatant was then loaded onto an MBPTrap HP 1 mL column (Cytiva, USA) that had been pre-equilibrated with the MBP Binding Buffer. Finally, purification was performed using the MBP Elution Buffer (10 mM maltose in MBP Binding Buffer), following the manufacturer’s instructions.

### Factor Xa Cleavage and Transmission Electron Microscopy

Transmission electron microscopy was used to determine whether VLPs can self-assemble from the expressed HPV16 L1 protein. Briefly, 60 μg of the purified MBP-HPV16 L1 was cleaved with 10 μg of Factor Xa Protease (Promega, USA) at room temperature for 15 h. The MBP-cleaved HPV16 L1 was applied to carbon support film and incubated at room temperature for 10 min. The sample was negative-stained with 2% phosphotungstic acid (PTA), and VLP formation was observed using a field emission transmission electron microscope (FE-TEM, Jeol, Japan) at 200 kV. Protein concentration was determined through the BCA method using a Pierce BCA Protein Assay Kit (Thermo Fisher Scientific).

## Results

### Construction of Recombinant *E. coli* Overexpressing MBP-HPV16L1

**Fig. 2:**
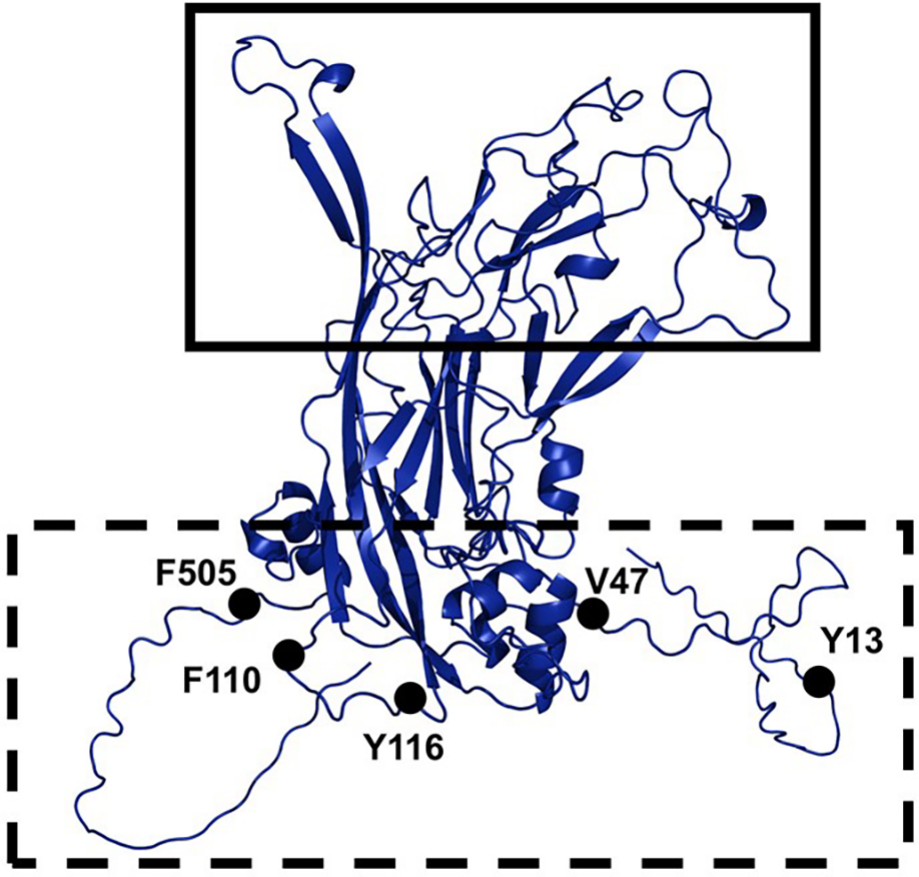
Three-dimensional structure (PyMol image) of HPV16 L1. The epitope site (solid square; top part) protrudes outward, while the bottom part (dashed square) faces inward during VLP formation. The five selected amber codon substitution sites are highlighted: Y13, V47, F110, Y116, and F505.

As demonstrated in Fig. 1, the pMALc2X-HPV16L1 was constructed by cloning the HPV16 L1 gene from pGST-opt-HPV into pMAL-c2X. Fig. 1D represents a collection of 5 recombinant pGoH-Amb plasmids, each harboring an amber codon substituted from the HPV16 L1 gene of pGST-opt-HPV. pMALc2X-HPV16L1/Amb represented in Fig. 1E was generated by subcloning each HPV16 L1 gene from the recombinant pGoH-Amb plasmids into pMAL-c2X. For the selection of amber sites in HPV16 L1 gene, the 3-dimensional structure of HPV16 L1 was analyzed using AlphaFold2 and PyMOL. Based on the analysis, five locations on the HPV16 L1 gene were selected, which would not affect the function and paricle formation (Fig. 2).

### Amber Suppression

**Fig. 3:**
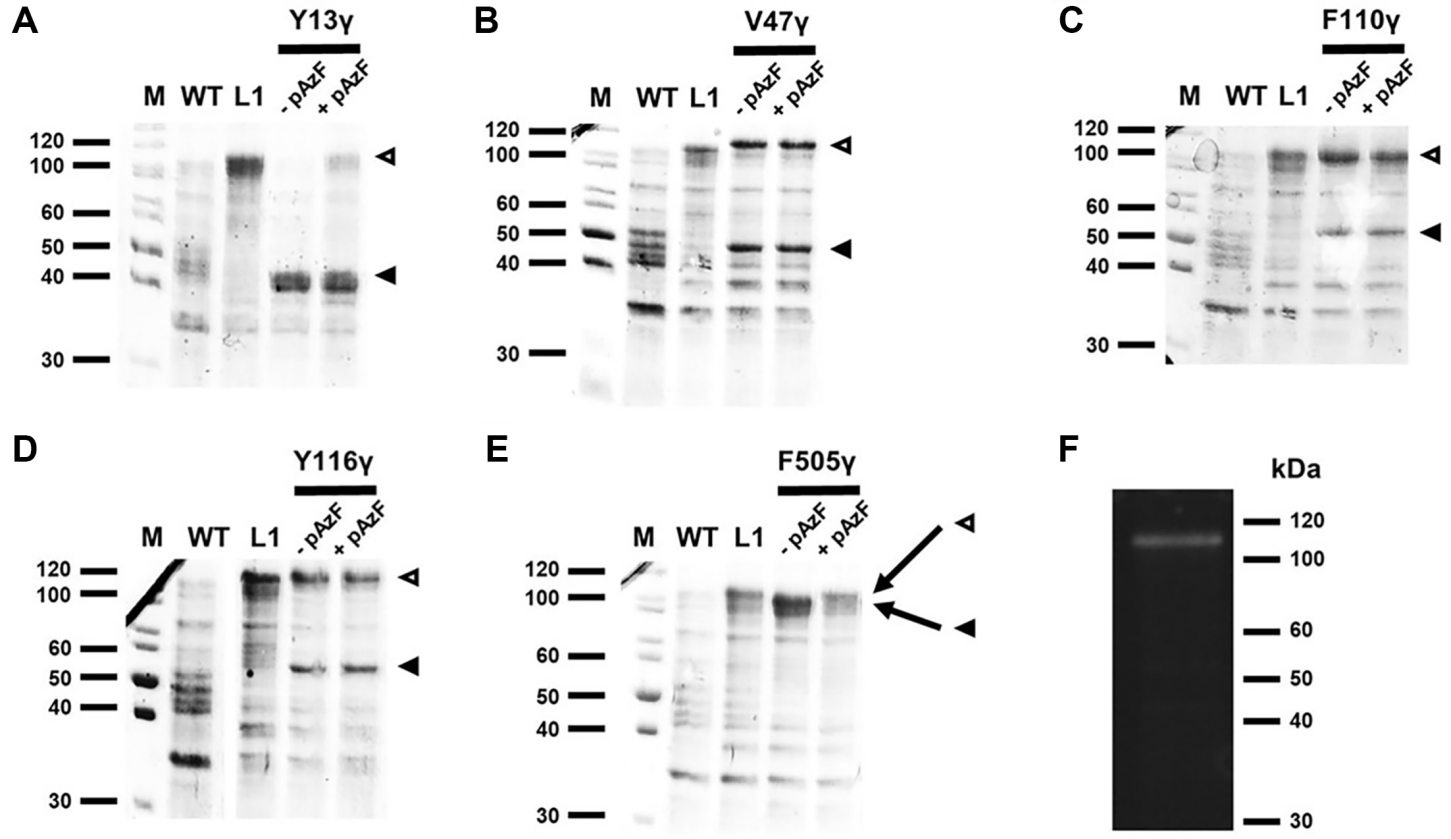
SDS-PAGE of pAzF-incorporated MBP-HPV16L1. Black arrows indicate bands corresponding to the truncated form of each MBP-HPV16L1 Amb candidate, while white arrows outlined in black indicate bands corresponding to pAzF-containing MBP-HPV16L1 proteins. M, molecular marker; WT, *E. coli* BL21 (DE3); L1, *E. coli* BL21 (DE3) pMALc2XHPV16L1; -pAzF, each induced candidate without pAzF; +pAzF, each induced candidate with pAzF. *E. coli* BL21 (DE3) (pMALc2X-HPV16L1/Amb1, pEVOL-pAzF) (**A**) *E. coli* BL21 (DE3) (pMALc2X-HPV16L1/Amb2, pEVOL-pAzF) (**B**) *E. coli* BL21 (DE3) (pMALc2X-HPV16L1/Amb3, pEVOL-pAzF) (**C**) *E. coli* BL21 (DE3) (pMALc2X-HPV16L1/Amb4, pEVOLpAzF) (**D**) and *E. coli* BL21 (DE3) (pMALc2X-HPV16L1/Amb5, pEVOL-pAzF) (**E**) respectively. (**F**) Fluorescence image from MBP-HPV16L1 F505γ conjugated with DBCO-PEG_3_-FITC.

Recombinant *E. coli* BL21 (DE3) harboring pMALc2X-HPV16L1/Amb candidates were transformed with pEVOL-pAzF, and these recombinants were overexpressed in the presence or absence of pAzF. The lysates of five MBP-HPV16L1 Amb candidates were compared using SDS-PAGE (Fig. 3). In this study, the HPV16 L1 protein with pAzF incorporation was named using the Greek letter γ. The predicted molecular weights of the recombinant MBP-HPV16L1 Amb candidates were as follows: for constructs without pAzF, MBP-HPV16L1 Y13γ, 44.5 kDa; MBP-HPV16L1 V47γ, 48.5 kDa; MBP-HPV16L1 F110γ, 55.6 kDa; MBP-HPV16L1 Y116γ, 56.3 kDa; MBP-HPV16L1 F505γ, 99.5 kDa; whereas the predicted molecular weight of constructs with pAzF was approximately 102.4 kDa. It was confirmed that V47γ (Fig. 3B), F110γ (Fig. 3C), and Y116γ (Fig. 3D) showed no difference in expression with or without pAzF, while Y13γ (Fig. 3A) and F505γ (Fig. 3E) expression levels. However, only F505γ was successfully expressed in its mature form with pAzF incorporated.

To confirm the incorporation of pAzF in HPV16 L1 protein, the overexpressed MBP-HPV16L1 F505γ was reacted with the DBCO-PEG_3_-FITC linker to verify whether pAzF was incorporated into HPV16 L1. The conjugation products were separated through SDS-PAGE, and UV visualization confirmed that pAzF was successfully incorporated into HPV16 L1 (Fig. 3F).

### Formation of VLPs

**Fig. 4:**
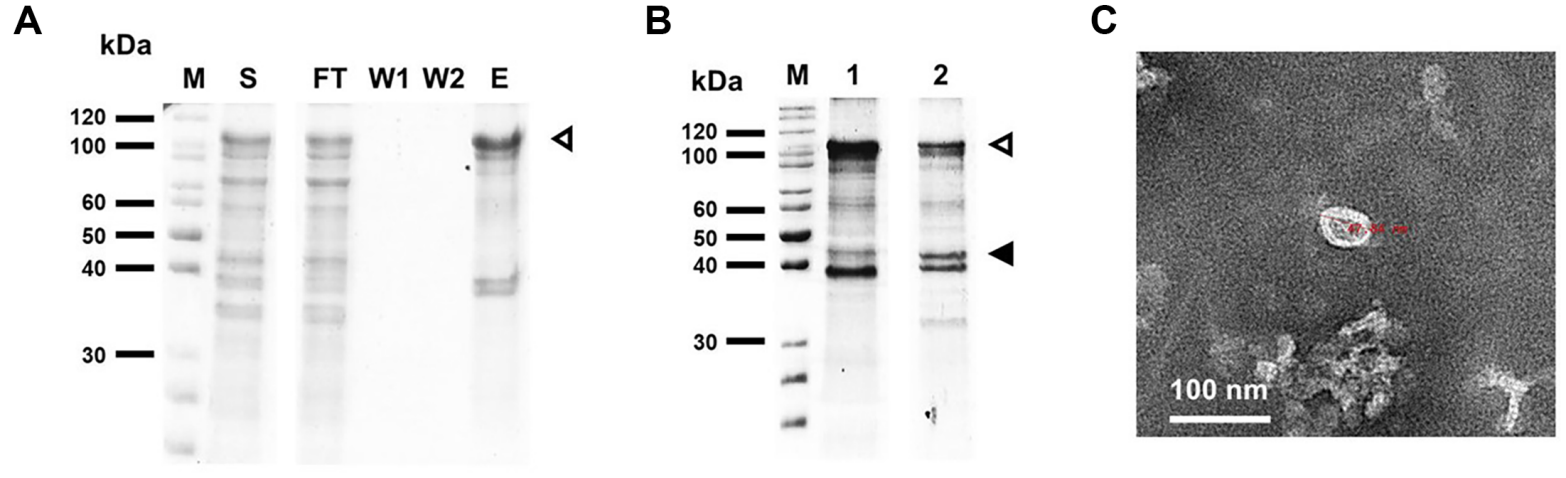
Analysis of VLPs formation. (**A**) Purification of MBP-HPV16L1. The white arrow outlined in black indicates the band corresponding to purified MBP-HPV16L1. M, molecular marker; S, supernatant fraction; FT, sample flow-through; W1, 1^st^ column washing step with the MBP binding buffer; W2, 2^nd^ column washing step with the MBP binding buffer; E, protein elution using the MBP elution buffer. (**B**) Cleavage of MBP-HPV16L1 using Factor Xa. The black arrow indicates the band corresponding to cleaved MBP, while the white arrow outlined in black indicates the band corresponding to purified MBPHPV16L1. M, molecular marker; lane 1, purified MBP-HPV16L1; lane 2, MBP-HPV16L1 cleaved using Factor Xa. (**C**) TEM images of MBP-cleaved HPV16 L1 protein. Samples were stained with 2% PTA and photographed at a ×50,000 magnification.

To investigate the self-assembly of HPV16 L1 and HPV16L1 F505γ, proteins were expressed and purified. First, to express MBP-HPV16 L1, the pMALc2X-HPV16L1 construct was transformed into *E. coli* BL21 (DE3), and the overexpressed proteins were analyzed using SDS-PAGE. The SDS-PAGE results of soluble lysate confirmed the successful expression of HPV16 L1 in its MBP-fused form. The supernatant containing MBP-HPV16L1 was purified (Fig. 4A), and the final concentration of purified MBP-HPV16L1 protein was about 4 mg/l culture.

To release HPV16 L1 from the MBP tag, purified MBP-HPV16L1 was cleaved with Factor Xa (Fig. 4B). After cleavage, the sample was negatively stained and analyzed using TEM. Despite the low yields, VLP structures with diameters of approximately 3 – 40 nm were observed (Fig. 4C). HPV16L1 F505γ was also expressed and soluble form fused with MBP tag was confirmed. After purification and cleavage, the VLP strutures were also observed. In previous study, HPV16 L1 expressed in *E. coli* formed small-sized VLPs approximately 30 nm in diameter composed of 12 pentamers in a T=1 arrangement [6]. Crystallographic analysis confirmed that the distances between the pentamer tips were sufficient to facilitate IgG bridging, comparable to T=7 VLPs. The result in this study indicates that the expressed HPV16 L1, without deletion of amino acids, can successfully self-assemble in vitro.

## Discussion

HPV is the primary cause of cervical cancer, one of the most prevalent cancers in humans. The production and purification of the L1 protein, the major capsid protein of HPV, are crucial for the development of HPV vaccines. While numerous studies have focused on production of the L1 protein using various expression systems, few have focused on the intrinsic properties of the L1 protein itself [11, 34-36]. In this study, we aimed to produce an modified HPV16 L1 by incorporating pAzF, a non-canonical amino acid, in *E. coli*.

MBP tag is one of the fusion partners that can be employed to enhance the solubility of co-expressed proteins. There has been reported that MBP tag functions similar to a molecular chaperone, significantly increasing the solubility of co-expressed proteins compared to GST tag [37]. HPV16 L1 protein was overexpressed in *E. coli* with the plasmid pGST-opt-HPV [37]. However, the protein was expressed in the insoluble form and difficult to be purified. Therefore, in this study, HPV16 L1 was fused with MBP tag and expressed in a soluble form.

pAzF is one of the noncanonical amino acids, and generally used for click chemistry. The azide group in pAzF can specifically react with an alkyne group via the strain-promoted azide-alkyne cycloaddition (SPAAC) to form a triazole structure. SPAAC allows the pAzF-incorporated protein to be used for protein modifications such as albumination [15]. To verify the expression of pAzF-incorporated HPV16 L1, we substituted a total of five locations in HPV16 L1 with amber codon to determine the expression in the presence or absence of pAzF. The expression results showed that MBP-HPV16L1 V47γ, MBP-HPV16L1 F110γ, and MBP-HPV16L1 Y116γ with the full-length HPV16 L1 was observed even without pAzF. It meant that these proteins did not incorporate pAzF at specified amber sites. Because the mutant aminoacyl-tRNA synthetase (aaRS) used in this study did not completely suppress these amber sites, the full-length proteins without pAzF could be expressed. HPV16L1 Y13 γ was confirmed with amber suppression, but expression of the full-length HPV16 L1 with pAzF was relatively low. Notably, proper amber suppression and expression level were only achieved when pAzF was incorporated at F505 (Fig. 3). As shown in Fig. 2, the HPV16 L1 protein is composed of a core part (8-strand β-jellyroll) and invading arms at the N-terminal and C-terminal that interact with other neighboring HPV16 L1 proteins to form pentamer. The core part is complexly intertwined with several β-sheets and is also involved in determining the solubility of the protein. Among the amber codon sites introduced in our experiments, F505 differs from other amber codon sites in that it is located in the C-terminal arm of the HPV16 L1 protein. More specifically, Y13 and V47 are located at the N-terminal of the protein before the core part, and F110 and Y116 are located in the loop between the β-sheets.

When incorporating ncAAs into a protein, an orthogonal tRNA/aaRS pair is employed [29, 40]. However, the location of the ncAAs can affect the protein expression pattern due to potential misreading of tRNAs during translation or nonspecific incorporation of natural amino acids. Factors such as expression system, plasmid used, the structural complexity and characteristics of the protein, activity of the tRNA/aaRS pair, and codon usage significantly affect amber suppression [18, 38, 39]. Therefore, the specific pattern of amber suppression induced by pAzF, which possesses a large aromatic ring, into a protein can only be determined through experimentation. In our study, we strategically selected the amber suppression site based on previously outlined reasons, in addition to considering pAzF’s structural similarity to tyrosine and phenylalanine. Consequently, only the MBP-HPV16L1 F505γ successfully induced full-length protein expression, as confirmed using the DBCO-PEG_3_-FITC linker (Fig. 3).

The ability of HPV L1 to self-assemble into VLPs emphasizes its potential as an HPV vaccine. These HPV VLPs without a viral genome can induce immune responses against HPV. The utilization of L1-based VLPs in HPV vaccines is well-established [5, 41]. Therefore, the confirmation of VLP formation can indicate the feasibility of VLPs as a vaccine candidate against HPV. The pAzF incorporation would not affect the antigenic properties of the HPV16 L1 protein. The antigenicity of L1 protein depends on the interaction of the FG loop and HI loop on the top surface of the capsomer knob shown in Fig. 2 with IgG antibodies that neutralize HPV. The location of pAzF on MBP-HPV16L1 F505γ protein is distant from the epitope region. Therefore, it is not expected to affect the interaction of the epitope region with the IgG antibody during the formation of VLPs.

The incorporation of pAzF into proteins can enhance their properties, enabling modifications such as albumination and PEGylation. In future experiments, the conjugation of human serum albumin with recombinant pAzF-incorporated HPV16 L1 will proceed to evaluate the utility as an HPV vaccine with enhance characteristics. This study is significant as it demonstrated that recombinant proteins with non-canonical amino acids for vaccine development could be produced in *E. coli*.

## Figures and Tables

**Table 1:** Details of the plasmids, primers, and strains used in this study.

Plasmids and strains	Characteristics	Source
Plasmid		
pGST-opt-HPV	pGEX-4X-1 derivative, tac promoter, lacI^q^, Amp^R^	[30]
pMAL-c2X	pMAL derivative, lacks the malE signal sequence, expresses proteins as MBP-fusion proteins in the cytoplasm	Addgene (ID: 75286)
pMALc2X-HPV16L1	pMAL vector harboring HPV16 L1 gene	This study
pGoH_Amb1	pGST-opt-HPV vector with Y13UAG	
pGoH_Amb2	pGST-opt-HPV vector with V47UAG	
pGoH_Amb3	pGST-opt-HPV vector with F110UAG	
pGoH_Amb4	pGST-opt-HPV vector with Y116UAG	
pGoH_Amb5	pGST-opt-HPV vector with F505UAG	
pMALc2X-HPV16L1/Amb1	pMAL vector harboring HPV16 L1 gene on pGoH_Amb1	
pMALc2X-HPV16L1/Amb2	pMAL vector harboring HPV16 L1 gene on pGoH_Amb2	
pMALc2X-HPV16L1/Amb3	pMAL vector harboring HPV16 L1 gene on pGoH_Amb3	
pMALc2X-HPV16L1/Amb4	pMAL vector harboring HPV16 L1 gene on pGoH_Amb4	
pMALc2X-HPV16L1/Amb5	pMAL vector harboring HPV16 L1 gene on pGoH_Amb5	
pEVOL-pAzF	pEVOL plasmid harboring two copies of synthetase and one copy of tRNA for *para*-azido-*L*-phenylalanine	Addgene (ID: 31186)
Strain		
*E. coli* BL21 (DE3)	Gene cloning and expression host	New England Biolabs
Primers for the construction of pMALc2X-HPV16L1		
Primer	Primer Sequence	Target Gene
pMAL_HPV_FP1	TCGGATCCCAAGTTACCTTTATCTA	HPV16 L1
pMAL_HPV_RP1	CAAGCTTTTACAGTTTGCGTTTTTTG	
pMAL_HPV_FP2	AACTGTAAGCTTGGCACTGGCC	pMAL-c2X
pMAL_HPV_RP2	TAACTTGGGATCCGAATTCTGAAATC	
Primers for the construction of pGoH-Amb candidates		
Primer	Primer Sequence	Target Gene
pGoH_Amb_F1	TGTCAACGTCTATCATATCTTCTTC	pGST-opt-HPV
pGoH_Amb_R1	TCGTTTTCCTAGCAGGTAATG	
pGoH_Amb_F2	ATGAATACGTCGCCCGTA	
pGoH_Amb_R2	CGGTCGACTACACTTTTGA	
pGoH_Amb_F3	AATTTGGCTAGCCGGATAC	
pGoH_Amb_R3	TATTCGGGTCCGGCA	
pGoH_Amb_F4	CTCATTCTAGAACCCGGAC	
pGoH_Amb_R4	GTATCCGGGAAGCCAAATT	
pGoH_Amb_F5	GCAAACGCAAAGCCAC	
pGoH_Amb_R5	CCAGGGTCTATTTCGGTT	
Primers for the construction of pMALc2X-HPV16L1/Amb candidates		
Primer	Primer Sequence	Target Gene
pMAL_HPV_sub_FP1	GGATTTCAGAATTCGGATCCCAAGTTACCTTTA	Mutant HPV16 L1
pMAL_HPV_sub_RP1	GCCAGTGCCAAGCTTTTACAGTTTGCGTTT	

Substituted nucleotides for mutagenesis were underlined.
